# A survey of bullous diseases in a Turkish university hospital: clinicoepidemiological characteristics and follow-up

**DOI:** 10.3906/sag-2006-231

**Published:** 2021-02-26

**Authors:** Zekayi KUTLUBAY, Ayşegül SEVİM KEÇİCİ, Uğur ÇELİK, Cem MAT

**Affiliations:** 1 Department of Dermatology, İstanbul University-Cerrahpaşa, Cerrahpaşa Medical Faculty, İstanbul Turkey; 2 Department of Dermatology, University of Medical Sciences, Haydarpaşa Numune Training and Research Hospital, İstanbul Turkey; 3 Department of Dermatology, Medipol University, İstanbul Turkey; 4 Department of Dermatology, Private Practice, İstanbul Turkey

**Keywords:** Bullous diseases epidemiology, pemphigus vulgaris, bullous pemphigoid

## Abstract

**Background/aim:**

Autoimmune bullous diseases, if left untreated, are life-threatening conditions affecting primarily skin and mucous membranes. These blistering disorders are characterized by epidermal or subepidermal detachment. Autoimmunity plays a key role in pathogenesis; therefore, immunosuppressive agents are the treatment of choice. The aim of this study is to document relative frequencies of different autoimmune bullous diseases, patient characteristics, treatment options, and side effects in patients presenting to our bullous skin disease center at İstanbul University, Cerrahpaşa, Cerrahpaşa Medical Faculty.

**Materials and methods:**

Medical files were examined retrospectively for all patients with autoimmune bullous diseases who were followed up between 2003 and 2019 at the Bullous Skin Disease Center at İstanbul University, Cerrahpaşa.

**Results:**

A total of 346 patient files were examined. Pemphigus vulgaris was the most frequent autoimmune bullous disease, followed by bullous pemphigoid and pemphigus foliaceus, according to our study. There is a general female predominancy for all autoimmune bullous diseases. The most commonly preferred treatment options were high-dose daily corticosteroids.

**Conclusion:**

This retrospective study summarizes the patient characteristics, comorbidities, treatment choices, and side effects during 16 years of clinical practice.

## 1. Introduction

Autoimmune bullous diseases (AIBDs) are rare but potential­ly devastating disorders of the skin and mucous membranes, characterized by the presence of tissue-bound and circulating antibodies directed against disease-specific target antigens. Based on the level of blister formation, these diseases can be divided into 2 groups: intraepidermal immunobullous diseases, also referred to as the pemphigus group, and sub­epidermal immunobullous disorders. The pemphi­gus group comprises pemphigus vulgaris (PV) and its variant pemphigus vegetans, superficial pemphigus (pemphigus fo­liaceus (PF) and pemphigus erythematosus (PE)), paraneo­plastic pemphigus (PNP), and IgA pemphigus. The incidence of pemphigus ranges from 0.5 to 16.2/1,000,000 per year [1–5]. The sub­epidermal immunobullous disorders group includes pemphigoid diseases (bullous pemphigoid (BP), mucous membrane/cicatricial pemphigoid (MMP/CP), pemphigoid gestationis (PG), linear IgA disease (LAD), and lichen planus pemphigoides (LPP)), epidermolysis bullosa acquisita (EBA), dermatitis herpetiformis (DH), and bullous systemic lupus erythematosus (BSLE). The incidence of BP has been estimated between 2 and 42.8/1,000,000 per year [1,4,6,7]. 

PV is frequently observed in Turkey, as in other countries of the Mediterranean region. However, there has not yet been any study of the relative frequencies and demographic features of different AIBDs in Turkey. Notably, the relative frequencies of sub­epidermal immunobullous disorders versus those of diseases in the pemphigus group are unknown. Our aim is to define the spectrum of AIBDs seen at the Center for Bullous Diseases in the Cerrahpaşa Medical Faculty, one of the major tertiary referral centers for AIBDs in Turkey, over a 16-year period.

## 2. Materials and methods

A retrospective analysis was performed at the Bullous Disorders Center in the Department of Dermatology and Venereology, University Hospital of Cerrahpaşa Medical Faculty in İstanbul, which is one of the major tertiary care follow-up and referral centers for patients with bullous diseases in Turkey. Although there is no systematic referral system or central registry for AIBDs in Turkey, this hospital is one of the most preferred tertiary referral centers, both in terms of public and private hospitals. 

All medical files of newly diagnosed patients with AIBDs were retrospectively recruited and analyzed for a period of 16 years between 2003 and 2019. Diagnoses were based on clinical findings, histo­pathology of affected skin or mucosa, direct immunofluore­scence (DIF) microscopy of perilesional mucous membrane or skin biopsies, indirect immunofluorescence (IIF) microscopy on monkey esophagus, and enzyme-linked immunosorbent assay (ELISA) for anti-desmoglein 1 and 3 antibodies. Patient inclusion criteria for the retrospective analysis comprised a diagnosis of AIBD confirmed by histopathological and direct immunofluorescence (DIF) examinations of tissue samples, in addition to clinical findings. Thus, histopathological examination and direct and indirect immunofluorescence examination were performed for all the patients included in this study. 

Detailed reviews of the patients’ clinical histories, other autoimmune diseases, and comorbidities of patients and their relatives, in addition with data about age, sex, age at onset of the disease, and duration of the disease were recorded. Clinical status at the onset such as mucosal and/or cutaneous involvements was evaluated. Histopathological examination, direct and indirect immunofluorescence test results, ELISA testing of antidesmoglein (anti-Dsg) 1 and 3 antibodies for the pemphigus group, local and systemic treatment modalities, relapses and remissions, side effects, and reported deaths during the follow-up period were also recorded.

## 3. Results

Between 2003–2019, 346 new cases of AIBDs, 151 men and 195 women (an average of 23 new cases per year), were analyzed in this study. The average annual age-adjusted AIBD incidence rate for our center was 0.307 per million in the population for both sexes. The mean age was 54.4, and mean duration of the disease was 85.6 months. Only 5 of all of these patients had a family history. The most common diagnoses were PV, BP, and PF, respectively (70%, 13%, and 6%), all of which seem to have occurred more commonly in females. Other rare diagnoses included hereditary epidermolysis bullosa, pemphigoid gestationis, Hailey–Hailey disease, dermatitis herpetiformis, subcorneal pustular dermatosis, LPP, and Grover’s disease. Table 1 summarizes the epidemiological and clinical characteristics of all AIBDs. 

**Table 1:** Epidemiological and clinical characteristics of all autoimmune bullous disease patients.

	RF	F/M	FH	MA	MD	M	C	M&C
PV	242	1.4	1	51.4	77.9	77	30	135
BP	45	1.25	-	75.6	75.4	1	35	9
PF	23	1.09	-	49.4	84	-	23	-
LAD	13	1.6	1	43.7	77	-	12	1
EBA	7	0.75	1	49.4	298	-	4	3
PE	5	-	1	57	171	-	5	-
MMP	4	1	-	67	75	4	-	-

RF: Relative frequency; F/M: Female to male ratio; FH: Family history (n); MA: Mean age (years); MD: Mean duration of disease (months); M: Only mucosal involvement; C: Only cutaneous involvement; M & C: Mucosal and cutaneous involvement.

The mean follow-up period for PV patients was 79.9 months, whereas for PF it was 82.8 months and for BP the total was 75.4 months. The most commonly used treatment options were high-dose corticosteroids and immunosuppressives such as azathioprine, mycophenolate mofetil, or sodium. Plasmapheresis, methotrexate, cyclosporine, dapsone, and colchicine in the case of acquired epidermolysis bullosa were less preferred options. Intravenous immunoglobulin, rituximab and pulse steroid therapy were commonly used in recalcitrant cases. For AIBDs, a high-dose daily systemic steroid was the most preferred treatment, with azathioprine being the most common adjuvant, as summarized, together with other treatment options, in Table 2. It is important to note that the most common adverse events such as osteoporosis, aseptic bone necrosis and compression fractures, myopathy, and cataracts are related to high-dose and long-term use of systemic corticosteroids. Other rare side effects include depression, diabetes, hypertension, neuropathy, pancytopenia, glaucoma, hepatotoxicity, pneumonia, and steroid acnes. To the best of our knowledge, a total of 8 deaths were declared during the follow-up period of these patients; however, there may have also been some underreporting. Three of the patients died due to bronchopneumonia, 3 from cardiopulmonary arrest, and the exact cause of death for the remaining 2 patients remains unknown. 

**Table 2:** Systemic treatment options for autoimmune bullous skin disorders.

	Patients (n)	SS	PS	Aza	MM	MS	IVIg	Rit	Dp	Mtx
PV	242	214	48	214	33	22	42	43	-	4
BP	45	-	1	10	-	-	-	-	6	1
PF	23	21	4	21	3	2	3	2	-	-
LAD	13	7	-	4	-	-	-	1	7	-
EBA	7	5	-	-	-	-	-	-	1	-
PE	5	4	-	4	-	-	-	-	-	-
MMP	4	3	-	1	-	-	-	-	-	-
Total	339	-	53	254	36	24	45	46	14	5

SS: Systemic steroid; PS: Pulse steroid; Aza: Azathioprine; MM: Mycophenolate mofetil; MS: Mycophenolate sodium; IVIg: Intravenous immunoglobulin; Rit: Rituximab; Dp: Dapsone; Mtx: Methotrexate.

### 3.1. Pemphigus vulgaris

Two hundred and forty-two patients were diagnosed as PV (242/346, 70%). Only one of them had a family history. The female to male ratio was 1.4 (f: 141, m: 101) and the mean age was 51.4 years. The mean duration of the disease was 77.9 months. Table 3 summarizes the comorbidities of PV patients, together with other AIBDs. Approximately 12% of all PV patients (30 patients) presented with only cutaneous lesions, and 32% (77 patients) of them with only mucosal lesions, while remaining 135 patients (56%) had both cutaneous and mucosal involvements. In addition to systemic treatment modalities, all PV patients received local treatment, including topical antimicrobials and local corticosteroid ointments. All patients with mucosal involvement were also given intralesional triamcinolone acetonide (20 mg/mL) injections. All PV patients were started on high-dose systemic corticosteroids immediately after diagnosis. The mean initial systemic steroid dosage was 100 mg/day of methylprednisolone or the equivalent, which gradually tapered to maintenance levels in a few months to years, according to the clinical course and prognosis of each and every patient. Azathioprine was the most commonly used adjuvant, with a mean daily dosage of 150 mg. Mycophenolate mofetil (2000 mg/day) or mycophenolate sodium (1440 mg/day) were the second-line adjuvants and were preferred in patients who could not tolerate azathioprine or had a long-term history of azathioprine use. Pulse steroid therapy was given intravenously in divided doses for 3 consecutive days (1000 mg methylprednisolone per day). It was preferred in only 48 patients during this 16-year period in case of frequent relapses or to avoid side effects of long-term systemic steroids. 

**Table 3:** Most common comorbidities of pemphigus vulgaris, bullous pemphigus, pemphigus foliaceus, and pemphigus erythematosus patients.

Comorbidities	PV patients (n)	BP patients (n)	PF patients (n)	PE patients (n)
Hypertension	17	13	1	1
Diabetes mellitus	10	6	-	1
Hyperlipidemia	6	2	-	-
Osteoporosis	6	1	-	-
Hepatitis	2	2	-	-
Coronary heart disease	2	2	-	1
Cataract	2	4	1	-
Chronic renal failure	1	2	-	-
Hyperthyroidism	1	-	-	-
Benign prostate hyperplasia	2	2	-	1
Rheumatoid arthritis	2	1	1	-
Pulmonary tuberculosis	1	3	-	-
Alzheimer’s disease	-	3	-	-
Parkinson disease	-	1	-	-

Beside physical examination of all skin and mucosal membranes, follow-up examinations every 3 months included indirect immunofluorescence titer and antidesmoglein 1 and 3 antibody levels. Complete blood count, fasting blood glucose level, and liver and kidney function tests are also routinely done for detecting possible adverse events related to treatment. Annual bone mineral density measurements were also performed. Approximately 41% of all our patients had at least one relapse during their long-term follow-ups. The most common treatment-related adverse events are shown in Table 4. Throughout the follow-up period, 3 deaths were reported. 

**Table 4:** Most common treatment-related adverse events of pemphigus vulgaris and bullous pemphigoid patients.

Adverse events	PV patients (n)	BP patients (n)
Osteoporosis	36	4
Hepatotoxicity	13	3
Myopathy	9	2
Cataract	9	5
Diabetes	4	3
Oral candidiasis	3	1
Pancytopenia	3	2
Pneumonia	2	3
Neuropathy	2	-
Avascular necrosis	2	1
Deep vein thrombosis	1	-

#### 3.1.1. IVIg and rituximab therapy in recalcitrant pemphigus vulgaris

IVIg was used at a monthly dose of 2 g/kg, which was divided equally over 5 consecutive days for 3 to 9 months for refractory PV, alone or in combination with rituximab. Anti-CD20 monoclonal antibody rituximab was administered as 1000-mg intravenous infusions, 2 weeks apart. In our clinic, 59 patients used IVIg and/or rituximab, 55 of which had PV and 4 had PF. Fourteen patients used rituximab alone, while 13 patients used IVIg alone, and the remaining 32 patients used both agents in combination. In the ‘only IVIg’ group, 10 out of 13 patients had clearance of disease with no recurrence after a mean treatment duration of 4.3 months. Two patients were able to recover with IVIg (3 and 5 administrations, respectively), but they had recurrences 3 years after the treatment period, while the other patient still had ongoing disease reoccurrence in spite of 6 treatment courses of IVIg administration. In the ‘only rituximab’ group, 13 out of 14 patients had no recurrence after 1 or 2 administrations (2–4 grams in total). One patient was lost to follow-up. Out of 32 patients who were using combined therapy, 26 had no recurrences after a mean duration of 4.2 months. Three patients had only IVIg treatment for 6 months with limited response and, after the combination with rituximab, they healed completely. Two patients had reoccurring symptoms after 1 year of 3 IVIg and one rituximab course, but later on they responded well to the same treatment regimen. The remaining patient had refractory disease even after 6 courses of IVIg and one course of rituximab treatment. It is important to note that 1 patient had an episode of deep venous thrombosis during the 3rd session of IVIg infusions, which led to the discontinuation of the therapy.

### 3.2. Bullous pemphigoid

BP was the 2nd most frequent AIBD in our study, with 45 patients (13%). Among these patients, 35 had only cutaneous lesions, one of them had only mucosal lesions, while the remaining 9 patients had both. None of them had a family history. The female to male ratio was 1.25 (f: 25, m: 20), and the mean age of and mean duration of the disease were 75.6 years and 75.4 months, respectively. It is important to note that BP patients were significantly older than PV patients (mean age: 75.6 years vs. 51.4 years) with more comorbidity, as shown in Table 3. All patients received topical treatment as a first-line therapy. Lower systemic steroid doses (about 0.5 mg/kg/day) are preferred when compared to PV, with a mean initial dose of 55.4 mg/day of methylprednisolone; tapering to maintenance levels was done more rapidly. One patient was given due to long-term side effects of corticosteroids. Azathioprine (N: 10), dapsone (N: 6), pulse steroid therapy (N: 1), and methotrexate (N: 1) were adjuvants used for refractory cases. The main treatment-related side effects in this patient group are summarized in Table 4. Throughout the follow-up period, 6 recurrences and 5 deaths were reported. 

### 3.3. Pemphigus foliaceus

PF was the 3rd most frequent AIBD, with 23 patients. None of them had a family history. The female to male ratio was 1.09 (f: 12, m: 11), the mean age was 49.4, and the mean duration of the disease was 84 months. All of the patients had only cutaneous involvement, as expected. Two patients were treated with only topical corticosteroids, due to limited disease, and the remaining 21 patients were given high-dose systemic steroids, together with azathioprine. Two of them were switched to mycophenolate sodium and 3 to mycophenolate mofetil due to side effects. Three patients received IVIg, 1 patient received rituximab, another was given both, and 4 patients received pulse steroids, due to resistant disease, with successful results. Ten patients had recurrent attacks during the course of the disease. 

### 3.4. Other autoimmune bullous skin diseases

One patient was diagnosed with benign familial pemphigus and treated with topical agents. Four patients (2 males and 2 females) with mucous membrane pemphigoid were also treated as PV. Thirteen patients were diagnosed as having linear IgA disease. Only one of them had mucosal involvement, while the remainder had cutaneous lesions. Only one patient had a family history of the disease. The mean age of the patients was 44.5 years. One patient had ulcerative colitis, which is the most common disorder associated with linear IgA bullous dermatosis. Seven patients were given a combination of dapsone (50–100 mg/day) and systemic steroids, 3 patients used azathioprine, 1 patient was treated with colchicine, and 1 patient with rituximab. Of 8 epidermolysis bullosa patients, 5 received systemic corticosteroids, 1 was given dapsone, 1 received colchicine, and 1 recalcitrant case was treated with IVIg. 

## 4. Discussion

AIBDs are a group of rare and potentially lethal disorders characterized by the onset of vesiculobullous lesions on the skin or mucous membranes, alteration of cutaneous or mucous membrane components, and presence of pathogenic autoantibodies targeting structural proteins of the desmosomes and the dermal-epidermal junction. Most cases of AIBD occur sporadically, without evidence of geographic or familial clustering. Little is known about the epidemiology of AIBD in Turkey. Epidemiologic surveys encompassing the whole spectrum of AIBDs, not a single disease or a group of diseases, are also limited in other countries. This is the first study on the entire group of AIBDs in Turkey, based on strict diagnostic criteria, including clinical, histological, and immunohistological evaluation. The large number of patients and long observation pe­riod, coupled with the epidemiological and clinicopathological aspects, treatment modalities, and side-effect profiles, are the main assets of this study. On the other hand, the incidence of BP and DH could have been underestima­ted due to their atypical and mild clinical presentation in some individuals. There is an overall female predominance in AIBDs, and this female predominance also applied to individual diseases, except IgA pemphigus, LAD, and EBA, which involved only a few patients [8]. 

### 4.1. Pemphigus vulgaris 

PV is characterized by autoantibodies working against intercellular adhesion molecules: Dsg3 or both Dsg 1 and 3, resulting in suprabasal acantholytic blisters. Our present observation confirms the data in previous studies and adds further support to the earlier notion that the clinical phenotype of pemphigus correlates with the anti-Dsg autoantibody profile. As expected, PV was the most common type of AIBD in our study, representing 70% of all cases. This finding is in line with reports from other Mediterranean, Middle Eastern, and East Asian countries such as Kuwait [6], Iran [8], China [9], Malaysia [10], and also from other studies from Turkey [11] but contrasts with reports from countries such as Switzerland, Germany, the UK, and Singapore, where SABDs, notably BP, predominated [1,4,12,13]. Compared with epidemiologic surveys in different re­gions of the world, the incidence of PV in İstanbul is very similar to that of neighboring countries such as Macedonia [2], Romania [3], Bulgaria [14], and Kuwait [6]. While the incidence is higher than in Western European countries such as Switzerland and the United Kingdom [1,12,15–17], it is lower than the rate in Iran [5,18], Tunisia [17], Greece [19], as well as rates found in Jewish individuals in the US [20]. The incidence of PV has been estimated to be 0.76, 0.77, and 1.7 in terms of new cases per million people per year in Finland, Germany, and France, respectively [12,16,17]. In contrast, in countries around the Mediterranean sea, the reported incidence is significantly higher, with 6, 6.7, and 8 new cases per million people per year in Eastern Sicily, Tunisia, and Northern Greece, respectively [17,19,21]. In a study from Germany, the age-adjusted incidence of PV was 9-fold higher in patients with a migration background, compared with native Germans, emphasizing the geographical and thus genetic background of the disease [22]. Of all the pemphigus group patients, PV was the most common subgroup in our study, with a 90% incidence rate (PF 8% and PE 1,44%, the least frequent form of pemphigus). Our findings on PV being the most frequent type are also compatible with the rates of most other countries such as India [23], South Korea [24], Bangladesh [25], Saudi Arabia [26], Kuwait [6], Iran [27] Israel [28], South Africa [29], Bulgaria [14], Serbia, Montenegro [30], Macedonia [2], Croatia [31], Greece [32], Italy [33], Germany [12], Spain [34], and France [17]. This was also true for the UK, Switzerland, and Singapore [1,4,13]. On the other hand, there are some notable, exceptional regions such as Tunisia [17], Mali [35], South Africa [29], Brazil, and other Latin American countries [36,37] where fogo selvagem, an endemic form of PF predominated. In Finland [16], PE was the most common type of pemphigus and, in Morocco [38], equal frequencies of superficial and deep forms of pemphigus were observed. The mean age of 51.4 years old for PV patients in this report, as well as those in previous studies, is relatively low compared to mean ages reported in South Korea [24], Japan [39], and in most European countries where the disease is reported to present in the 6th and 7th decades of life. According to a study conducted in Serbia, when age-specific incidence rates are calculated, the incidence of PV among women (compared to men) remains higher until the age of 80 years or older, after which the incidence for men becomes slightly higher [40]. Our results of mean age are close to the reports from Iran [27], Saudi Arabia [26], South Africa [29], Singapore [41], and other studies conducted in Turkey [11]. However, the mean age of PV patients was lower than in our study group in Kuwait and India, with a presentation usually in the 4th decade of life [6,23]. In the present study, the number of women with PV outnumbered that of men (195 vs. 151, with a ratio of 1:4); which concurs with data from most previous studies worldwide, except for countries with a male predominance such as Saudi Arabia [26], Kuwait [6], Bangladesh [25], and China [9]. In this study, common comorbidities were hypertension, diabetes, hyperlipidemia, and osteoporosis, observed in 7%, 4%, 2.5%, and 2,5% of the patients, respectively. Frequent comorbidities for this patient population are cardiovascular diseases, concomitant malignancies, diabetes, and neurological disease, according to various studies [1]. 

High-quality controlled prospective clinical trials for the treatment of autoimmune blistering diseases are rare; therefore, treatment decisions are mainly guided by published reviews in this field or from personal experiences. In our clinical practice, all of the patients with PV were started on high-dose systemic steroids (1–1.5 mg/kg/day of methylprednisolone), together with at least one immunomodulatory adjuvant, most commonly azathioprine or mycophenolate, in order to minimize the adverse effects of steroid therapy. Pulse steroid therapy, rituximab, and IVIg are treatments of choice in case of relapse. Our mean initial systemic steroid dose in this study was 100 mg/day of methylprednisolone. However, especially in Western countries, treatment approaches are less aggressive, with lower doses of systemic steroids for shorter periods and occasionally with adjuvants [1]. This is probably due to the milder course of disease in these populations. In our patient group, 20% had at least 3 infusions of pulse steroid therapy, 11% used either rituximab or IVIg, and 13% used these 2 agents in combination. In total, almost half of our PV patients (44%) experienced relapses despite conventional steroids, in addition to adjuvant agents and required advanced treatment approaches for their recalcitrant disease. During the clinical course of PV, relapses are frequent and inevitable [42]. Almost half of the patient population (N: 100) had recurrent attacks. However, the rates of recurrence were significantly lower in the IVIg and rituximab groups (23% and 7%, respectively), similar to the literature [43]. It is important to consider the acute and chronic side effects of high-dose steroid infusions such as hypertension, hyperglycemia, fluid and electrolyte imbalances, sleep disturbances, mood alteration, and psychosis. Avascular necrosis of the hip, cardiac arrhythmias, cardiac arrest, and sudden death are rare but notable.

### 4.2. Pemphigus foliaceus 

In the case of PF, autoantibodies against desmoglein 1 result in superficial subcorneal blisters without mucosal involvement. PF was the 3rd most common AIBD, occurring at a frequency of 6.6%. It has been reported less frequently than PV worldwide, except in endemic areas of the world such as Brazil, North Africa, Tunisia, and Algeria [44]. A study [17] comparing the rate of incidence of PV in France and Tunisia showed a higher incidence rate in the former (6.7 vs. 1.7 new cases per million people per year), especially for PF occurring in young women. This is possibly related to exposure to risk factors that are either more prevalent in or more often expressed among young women living in particular areas of Tunisia. Another prospective study [45] found a significant association of PV occurring in young women from traditional Tunisian backgrounds who came into contact with ruminants, cut and handled raw poultry, had Turkish baths, or used cosmetics. The mean age and sex ratio of PF were close to those of PV in our study. (49.4 vs. 54.4)

### 4.3. Bullous pemphigoid

This ailment is characterized by autoantibodies against hemidesmosomes at dermoepidermal junction, and it presents with subepithelial blisters and, sometimes, urticarial plaques. In the present study, BP ranked 2nd in frequency among AIBDs, although it was much less common than PV (13% vs. 70%). The ratio of BP to PV has been reported to be 1:5.4, which is similar to ratios found in Singapore (3:1) and in a previous study conducted in Turkey (1:5.1) [11,13]. Unlike Turkey and other Eastern countries such as Kuwait, China, Iran, and Malaysia, this ratio is completely different in Europe [6,9,10]. 

Subepidermal AIBDs represent about 60% to 70% of all AIBD cases in Germany and France, with an incidence rate of 13.4 and 10.4 cases per year and per million inhabitants, respectively [22,46]. Similar results were also observed in Switzerland and the UK [1,4]. Compared to epidemiologic surveys in different parts of the world, the incidence of BP in Turkey is higher than that of neighboring Romania and Kuwait [6]. By contrast to the various studies mentioned above, the incidence of BP in different countries shows an opposite pattern, with 3–20 times higher values in Western European and Asian countries such as Germany, Italy, Switzerland, Scotland, Finland, Poland, the USA, Singapore, France, and the United Kingdom, where the incidence of BP is the highest (42.8 pmp/year) [1,4,12,13,47–54]. According to a very recent study, Serbia is the only country with similar average crude and age-adjusted incidence rates for both PV and BP [40]. 

The incidence rate of BP, especially in Western countries, progressively and significantly increases with age, particularly after the age of 70, with a peak after 90 years old [40]. The incidence rate appears to be higher in women until the age of 70 but, thereafter, the incidence is higher in men [1]. The mean age of presentation for BP in our study was 75.6 years old, which is significantly higher than PV, as expected. The lower incidence of BP and lower mean age of affected patients may reflect the paucity of the elderly population in less industrialized countries. Nevertheless, the possibility that different genetic or environmental factors may play some role in the disease profile cannot be ruled out. It must be noted that in most of these studies, only absolute numbers of patients were reported and not sex-specific incidence rates. In the present study, the highest incidence rates of pemphigoid for both sexes were registered in age groups 70–79 years old and particular­ly in individuals 80 years or older, which is in accordance with the literature. 

In our study, women were affected 1.25 times more often than men, which corresponds to results from other countries, except for Kuwait, which showed an even more pronounced female predominance (up to 6 times). Female predominance has been reported worldwide for BP, except in China and Germany [9,47]. Male predominance was also reported in the elderly population after adjusting for sex in another recent study from Germany [12]. 

The most frequently observed comorbidities in BP patients in our study were hypertension and diabetes mellitus, followed by Alzheimer’s disease at 29%, 13%, and 7% respectively, similar to other studies [55]. According to a study conducted in the United States, cardiovascular diseases were the most frequently associated health problem, affecting more than half of patients. Neurological diseases, malignancies, atopic diseases, chronic kidney failure, and diabetes were also common, similar to our cohort [56]. There are many studies in the literature establishing an association between BP and a higher prevalence of neurological disorders, namely dementia and cerebral stroke, when compared with the general population. In France, 36% of BP patients were diagnosed with at least 1 neurological disorder, namely dementia, in 20% of BP patients [57]. However, lacking a control population, it was not possible for this study to confirm or reject the association of BP with neurological diseases, especially with the cases of Parkinson’s disease, dementia, and multiple sclerosis reported in previous studies [58,59]. Chuang et al reported an association between BP and diabetes, showing 20% diabetic BP patients versus 2.5% diabetic controls (P = 0.004). This was supported by other studies. It has been proposed that an autoimmune response occurs after exposure to BP antigens by glycation of proteins of the dermoepidermal junction [60]. Considering the overall, advanced age of this population, the observed association between both cardiovascular and neurological diseases is not surprising. 

Notably, of the patients with BP, 71% were treated with systemic steroids only, and the mean initial corticosteroid dose required to control the disease symptoms was relatively lower than in the PV group (60 mg/day vs. 100 mg/day of methylprednisolone). The steroid dose was also tapered more quickly. In 17 cases (38%), systemic immunosuppressive drugs such as azathioprine, dapsone, and methotrexate were used as adjuvants. The main side effects observed during therapy were steroid-related, like osteoporosis and myopathy. 

### 4.4. Other bullous disorders 

MMP represented 0.86% of ABDs. This percentage may also reflect an underestimation of the incidence of this disease because many patients are diagnosed and seen by dentists and ophthalmologists.

EBA, in the group of mechanobullous disorders, was diagnosed in 2.02% of patients by DIF on salt-split skin. It was predominantly seen in men, as it was in Germany, Singapore, and China [9,12,13]. 

Thirteen patients (3.7%) were diagnosed as LAD. This finding may also reflect an underestimation, as we suppose that a significant proportion of such patients may have been diagnosed by pediatricians. Only 1 patient had a mucosal involvement, while the remainder had cutaneous lesions. Actually, this outcome is not compatible with studies found in the literature that say mucosal involvement occurs in up to 80% of patients [40]. One patient had accompanying ulcerative colitis, which is the most common disease associated with LAD. 

We were unable to accurately estimate the incidence and prevalence rates of bullous diseases in Turkey or in İstanbul according to the results of our study, although our clinic is the leading referral center for bullous diseases in the country. The lack of a central registry for bullous diseases and the limited number of other minor clinics or private practitioners covering the same patient population make this goal impractical at present. 

PV was the most frequent AIBD according to our study; cases of PV outnumbered those of BP by a ratio of almost 5:1. This contrasts with findings from Western European countries, where BP predominates. Female predominance was found in the patient profile for most subtypes of AIBD. The mean age at onset of PV and BP was lower in Turkey than in Europe and higher than in some Middle Eastern countries. Almost half of our PV patients were recalcitrant and required advanced treatment options, demonstrating the hard-to-control nature of the disease. Given the country’s large number of PV patients, future clinical trials regarding the efficacy and side effects of treatment options can be conducted in Turkey. Future studies may reveal additional information on patterns of incidence with respect to the entire spectrum of AIBDs in different areas of the world.

## Informed consent

The study protocol received institutional review board approval from ethical committee of Cerrahpaşa Medical Faculty and that all participants are provided informed consent.
